# Association between longitudinal memory performance and amyloid burden on PET scans in cognitive SuperAgers

**DOI:** 10.1002/alz70856_106210

**Published:** 2026-01-08

**Authors:** Daniel Gutstein, Molly A Mather, Nathan Pruneau Gill, Elena Barbieri, Jaiashre Sridhar, Allison Lapins, Sandra Weintraub, Robert J. Vassar, Changiz Geula, Marsel Mesulam, Tamar Gefen, Todd Parrish

**Affiliations:** ^1^ Mesulam Center for Cognitive Neurology & Alzheimer's Disease, Chicago, IL, USA; ^2^ Northwestern University Feinberg School of Medicine, Chicago, IL, USA

## Abstract

**Background:**

SuperAgers (SAs) are individuals aged 80+ with episodic memory performance comparable to those 20‐30 years younger. Preliminary examination of longitudinal neuropsychological performance suggests that, whereas many SAs maintain exceptional memory performance over time, some show gradual memory decline. Mechanisms of maintenance vs decline in SAs are not yet known but may include accumulation of the hallmark amyloid and tau pathologies of Alzheimer's disease. The current study investigated the association between PET amyloid burden in the brain and longitudinal episodic memory performance in SAs.

**Method:**

A total of 14 SAs enrolled in the Northwestern University SuperAging Program received a baseline PET amyloid scan (M_age_ = 84.88; 36% female). Individual memory trajectories were estimated from serial scores on the Rey Auditory Verbal Learning Test (RAVLT) delayed recall measure. The weighted mean for standardized uptake value ratio (SUVR), a quantitative measure of PET amyloid burden, was derived using the Susan Landau method and the FreeSurfer toolkit (v. 7); SUVR for all but one participant fell below the binary threshold for positivity. Linear mixed effects models accounting for PET tracer, sex, and age assessed the relationship between baseline SUVR and longitudinal memory performance.

**Result:**

Higher baseline SUVR was associated with a more negative longitudinal RAVLT slope (*p* < 0.05). However, this relationship was likely driven by the single participant with an elevated measure of PET amyloid burden (SUVR = 1.72), who also demonstrated a sharply negative slope of change in RAVLT score. When only individuals with SUVR below the positivity threshold were included, the relationship between baseline SUVR and slope of change in RAVLT over time was no longer statistically significant.

**Conclusion:**

Among SAs with SUVR measures of PET amyloid burden below the positivity threshold, variance in SUVR did not predict trajectory of change in memory performance over time. While accumulation of amyloid burden above known thresholds for PET positivity may increase risk for memory decline in SAs, this relationship was not apparent at lower levels of PET amyloid burden. Future study will investigate other in‐vivo markers of neurodegeneration to explore putative neuropathologic substrates of memory preservation vs decline in this unique population.

